# Slc6a20a Heterozygous and Homozygous Mutant Mice Display Differential Behavioral and Transcriptomic Changes

**DOI:** 10.3389/fnmol.2022.857820

**Published:** 2022-03-07

**Authors:** Junhyung Kim, Junyeop Daniel Roh, Seongbin Kim, Hyojin Kang, Mihyun Bae, Eunjoon Kim

**Affiliations:** ^1^Department of Biological Sciences, Korea Advanced Institute for Science and Technology (KAIST), Daejeon, South Korea; ^2^Center for Synaptic Brain Dysfunctions, Institute for Basic Science (IBS), Daejeon, South Korea; ^3^Division of National Supercomputing, Korea Institute of Science and Technology Information (KISTI), Daejeon, South Korea

**Keywords:** glycine and proline transport, NMDA receptors, autism spectrum disorders, epilepsy, learning and memory, synapse, mitochondria, ribosomes

## Abstract

SLC6A20A is a proline and glycine transporter known to regulate glycine homeostasis and NMDA receptor (NMDAR) function in the brain. A previous study found increases in ambient glycine levels and NMDA receptor-mediated synaptic transmission in the brains of *Slc6a20a*-haploinsufficient mice, but it remained unknown whether *Slc6a20a* deficiency leads to disease-related behavioral deficits in mice. Here, we report that *Slc6a20a* heterozygous and homozygous mutant mice display differential behavioral phenotypes in locomotor, repetitive behavioral, and spatial and fear memory domains. In addition, these mice show differential transcriptomic changes in synapse, ribosome, mitochondria, autism, epilepsy, and neuron-related genes. These results suggest that heterozygous and homozygous *Slc6a20a* deletions in mice lead to differential changes in behaviors and transcriptomes.

## Introduction

NMDA receptors (NMDARs) critically regulate the development and function of the nervous system (Paoletti et al., 2013; Zhu and Paoletti, 2015; Hansen et al., 2018). Glycine homeostasis is a key regulator of NMDAR function in the brain (Johnson and Ascher, 1987; Paoletti et al., 2013; Zhu and Paoletti, 2015; Hansen et al., 2018). Glycine levels in the extracellular spaces of the brain are thought to be regulated by two well-known glycine transporters, GlyT1 and GlyT2, which are encoded by Slc6a9 and Slc6a5, respectively (Smith et al., 1992; Liu et al., 1993).

SLC6A20A, which was originally reported to transport mainly proline (Smith et al., 1995; Nash et al., 1998; Kiss et al., 2002; Kowalczuk et al., 2005; Takanaga et al., 2005; Broer and Gether, 2012), was recently shown to also transport glycine and regulate glycine/proline homeostasis and NMDAR function in the mouse brain (Bae et al., 2021). Thus, SLC6A20A could be a novel target for inhibiting glycine uptake in the brain and thereby increasing ambient glycine levels and NMDAR function; this could be relevant for the treatment of NMDAR-related brain disorders (Bae et al., 2021), similar to the targeting of GlyT1 and GlyT2 for the treatment of schizophrenia, alcohol dependence, and pain (Tsai et al., 2004; Lane et al., 2006; Javitt, 2012; Harvey and Yee, 2013). However, it remains unclear whether deletion of *Slc6a20a* in mice leads to any disease-related behavioral deficits through specific mechanistic deviations.

In the present study, we show that *Slc6a20a* heterozygous and homozygous mice (*Slc6a20a*^+/–^ and *Slc6a20a^–/–^* mice, respectively) display differential behavioral deficits in the locomotor, repetitive behavioral, and memory domains as well as transcriptomic changes in genes associated with synapses, mitochondria, ribosomes, autism spectrum disorders (ASD), epilepsy, and neurons.

## Materials and Methods

### Mice

Mice lacking exon 3 of the *Slc6a20a* gene were previously described (Bae et al., 2021). All mice were maintained under a 12-hr light/dark cycle (light phase: between 1:00 a.m. and 1:00 p.m.), given food and water *ad libitum*, and weaned at postnatal day 21. Female heterozygous mice and male heterozygous mice were crossed in order to obtain wild-type, heterozygous, and homozygous progenies. All mice were maintained in the mouse facility of Korea Advanced Institute of Science and Technology (KAIST). The experimental procedures were approved by the Committee of Animal Research at KAIST (KA2020-89).

### Behavioral Tests

Adult male mice aged 3-6 months were used for all behavioral tests. All behavioral experiments were performed during the dark phase of the light/dark cycle (1:00 p.m. to 1:00 a.m.) in which mice are usually active. The brightness conditions in behavioral experiments described below refer to the local brightness around the center region of the apparatus rather than that in the behavioral room. Mice were habituated to an empty, dark experimental room for 30 min before the start of each behavioral test except for the Morris water maze test. EthoVision XT (Noldus) was used to analyze behavioral results unless otherwise noted.

### Open-Field Test

This experiment was performed in order to analyze the locomotor activity and anxiety-like behavior in mice. A mouse was placed into the center of a white box (40 cm x 40 cm x 40 cm; ∼100 lux in the center). Mouse movements were then recorded for an hour, and the distance moved and time spent in the center region of the apparatus were analyzed.

### Elevated Plus-Maze Test

This experiment was performed to investigate height-induced anxiety-like behavior in mice. A heightened, cross-shaped device with two arms closed and two arms open was used for the experiment. A mouse was placed in the center region of the apparatus (∼200 lux) and allowed to freely move around the environment for 10 minutes. Time spent in closed arms, open arms, and center was analyzed.

### Light-Dark Box Test

This experiment was performed to measure light-induced anxiety-like behavior in mice. The apparatus contained two different boxes/chambers, with one with the roof (dark box), and another without the roof (light box; ∼250 lux). A mouse was placed in the light box at the beginning and allowed to freely move around the environment.

### Three-Chamber Test

This test was performed to measure social approach and social novelty recognition (Crawley, 2004; Nadler et al., 2004). A mouse was placed in the center chamber of the three-chambered apparatus (59 cm x 39.5 cm x 21.5 cm; ∼100 lux), and its activity was recorded for 10 min. Then, a stranger mouse (S1) was positioned in the cage placed at the corner of a side chamber, and an object (O) was placed in the cage at the corner of the other side chamber. 129S1 mice from the Jackson Laboratory were used as strangers. After 10-min recording of mouse activity, the object was replaced with another stranger (S2), followed by 10-min recording.

### Morris Water Maze Test

This test was performed to measure spatial learning and memory in mice (Vorhees and Williams, 2006). A round pool was filled with white paint-added water. Water temperature was maintained at ∼20°C. The pool was divided into four quadrants, and a platform was positioned in one of the quadrants. For the first six days, mice were taught to find an invisible platform. On the seventh day, the probe test was performed: a platform was removed from the pool. A mouse was then placed in the center of the pool and allowed to freely swim around for a minute. Time spent in quadrants and number of crossings across the platform were measured. After the probe test, a platform was placed in the opposite quadrant. For additional three days, mice were trained to locate the new position of the platform (reversal phase). On the eleventh day, another probe test (reversal probe test) was performed.

### Contextual Fear Conditioning Test

This test was performed to measure fear learning and memory in mice. On day 0, a mouse was placed in the chamber with an electrocuting platform and habituated for 5 min. On day 1, foot shocks (0.8 mA) were provided for 2 s at 2, 3, and 4 min after the start of recording. Activity of a mouse was recorded for 5 min. On days 2, 3, and 8, a mouse was placed into the same chamber without any shock, and its activity was recorded for 5 min. Percent of freezing time was calculated.

### LABORAS

LABORAS (Laboratory Animal Behavior Observation Registration and Analysis System) experiments were performed for a long-term (4-day) monitoring of various mouse behaviors such as locomotor activity, repetitive behaviors (climbing, grooming, and rearing), drinking, and eating (Quinn et al., 2003, 2006). Total investigation time was 96 h with 12-h light/dark cycle, during which food and water were provided.

### RNA-Seq Analysis

Four mice at ∼P120 were used for each group (wild type, heterozygous, and homozygous). The extracted mouse brains were preserved in RNAlater solution (Ambion) and stored at −20°C. Poly-T oligo-attached magnetic beads were utilized to purify poly-A mRNAs. RNA concentrations were quantified using Quant-IT RiboGreen (Invitrogen, R11490), and RNA integrity was determined using TapeStation RNA screen tape (Agilent Technologies), after which only high-quality RNAs (RIN > 7.0) were selected for cDNA library construction using Illumina TruSeq mRNA Sample Prep kit (Illumina). Indexed libraries were submitted to an Illumina NovaSeq (Illumina), and paired-end (2 × 100 bp) sequencing was performed by Macrogen Inc.

Transcript abundance was estimated with Salmon (v 1.1.0) (Patro et al., 2017) in Quasi-mapping-based mode onto the *Mus musculus* genome (GRCm38) with GC bias correction (−gcBias). The acquired abundance data was imported to R (v.3.5.3) with tximport (Soneson et al., 2015) package and differential gene expression analysis was performed using R/Bioconductor DEseq2 (v1.30.1) (Love et al., 2014). Principal component analysis (PCA) was performed for the regularized log transform (rlog) of the normalized counts using plotPCA (with default parameter) tools implemented in DEseq2. Normalized read counts were computed by dividing the raw read counts by size factors and fitted to a negative binomial distribution. The *p-*values were adjusted for multiple testing with the Benjamini-Hochberg correction. Genes with an adjusted *p*-value of less than 0.05 were considered as differentially expressed. Volcano plots were generated using R ggplot2 (v.3.1.1) package. The Gene Ontology (GO) enrichment analyses were performed using DAVID software (version 6.8) (Huang da et al., 2009). Mouse gene names were converted to human homologs using the Mouse Genome Informatics (MGI) database. Gene Set Enrichment Analysis (GSEA) (Subramanian et al., 2005, 2007) was performed to determine whether *a priori-*defined gene sets would show statistically significant differences in expression between WT and *Slc6a20a*-mutant mice. Enrichment Analysis was performed using GSEAPreranked^®^ (gsea-3.0.jar) module on gene set collections downloaded from Molecular Signature Database (MSigDB) v 7.0. GSEAPreranked was applied using the list of all genes expressed, ranked by the fold change and multiplied by the inverse of the *p-*value with recommended default settings (1,000 permutations and a classic scoring scheme). The false discovery rate (FDR) was estimated to control the false positive finding of a given normalized enrichment score (NES) by comparing the tails of the observed and null distributions derived from 1,000 gene set permutations. The gene sets with an FDR of less than 0.05 were considered as significantly enriched. Integration and visualization of the GSEA results were performed using the EnrichmentMap Cytoscape App (version 3.8.1) (Merico et al., 2010; Isserlin et al., 2014).

### Statistical Analysis

GraphPad Prism (version 9.2.0; GraphPad Software) were used to perform the statistical analyses. Outliers were retained. Statistical details are presented in Supplementary Table 1.

## Results

### *Slc6a20a* Deficiency in Mice Induces Moderate Hyperactivity Without Affecting Anxiety-Like Behavior

Because glycine levels and NMDAR functions are elevated in *Slc6a20a*-mutant mice (Bae et al., 2021), and NMDARs critically regulate brain development and function (Paoletti et al., 2013), we subjected *Slc6a20a* heterozygous and homozygous mutant mice (*Slc6a20a*^+/–^ and *Slc6a20a^–/–^* mice, respectively) to a battery of behavioral tests.

**FIGURE 1:**
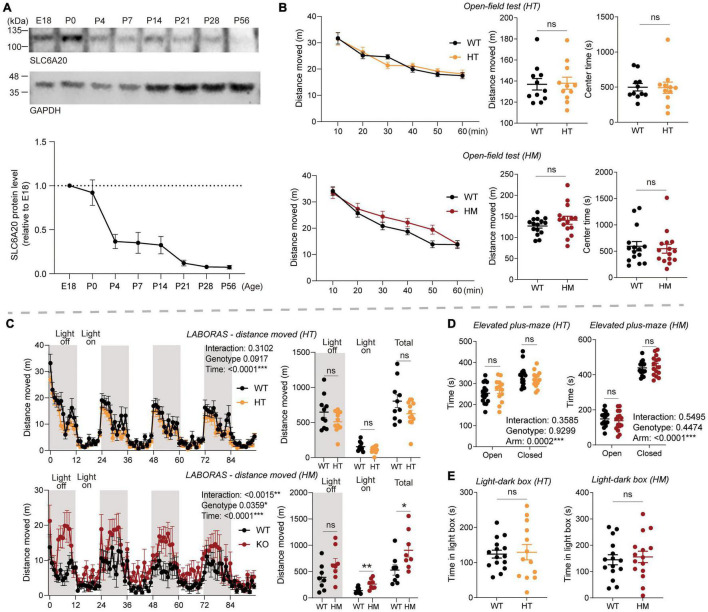
*Slc6a20a* deficiency in mice induces moderate hyperactivity without affecting anxiety-like behavior. **(A)** Temporal expression pattern of SLC6A20A proteins in mouse brains at the indicated embryonic (E) and postnatal (P) days. Whole-brain lysates were immunoblotting with SLC6A20 and control (GAPDH) antibodies. SLC6A20 signals were normalized to GAPDH signals for quantification. Note that the utilized pan-SLC6A20 antibody could recognize both SLC6A20A and SLC6A20B proteins in the brain; the expression level of SLC6A20A seems to be about three times greater based on the decrease in the pan-SLC6A20 signal of *Slc6a20a* homozygous mutant mouse brain (Bae et al., 2021) (*n* = 4 mice). **(B)** Locomotor activity in the open-field test is normal in *Slc6a20a*^+/–^ and *Slc6a20a^–/–^* mice (3–6 months; HT and HM, respectively) compared with WT mice, as shown by distance moved. Note that there is no genotype difference in the time spent in the center region of the open-field area, suggestive of normal anxiety-like behavior. (*n* = 11 mice [WT for HT], 11 [HT], 15 [WT for HM], 15 [HM], two-way repeated-measures/RM-ANOVA [distance moved] and Student’s *t*-test [total distance moved, time in center]). **(C)** Locomotor activity is moderately increased in *Slc6a20a^–/–^* but not *Slc6a20a*^+/–^ mice in the Laboras test, where mouse movements were measured for 4 consecutive days. Note that *Slc6a20a^–/–^* mice show hyperactivity during the total and light-on periods but not the light-off period, suggestive of disturbed sleep. (*n* = 10 mice [WT for HT], 12 [HT], 8 [WT for HM], 8 [HM], two-way RM-ANOVA [distance moved across 4 days] and Student’s *t*-test [total distance moved during total, light-off, and light-on periods]). **(D)** Anxiety-like behavior is normal in *Slc6a20a*^+/–^ and *Slc6a20a^–/–^* mice (3–6 months) in the elevated plus-maze test, as shown by time spent in the open/closed arms. (*n* = 16 mice [WT for HT], 13 [HT], 15 [WT for HM], 15 [HM], Student’s *t*-test). **(E)** Anxiety-like behavior is normal in *Slc6a20a*^+/–^ and *Slc6a20a^–/–^* mice (3–6 months) in the light-dark test, as shown by time in the light chamber. (*n* = 15 mice [WT for HT], 13 [HT], 15 [WT for HM], 15 [HM], Student’s *t*-test). Statistical significance and *p* values in the graphs; *p < 0.05, **p < 0.01, ***p < 0.001, ns, not significant.

As the first step, we used immunoblot analysis to determine the temporal pattern of Slc6a20a protein expression during brain development. The immunoblot pattern from whole-brain lysates indicated that protein expression was strong at early developmental stages, including late embryonic and neonatal stages, and thereafter decreased to adult levels over the first few weeks of postnatal life (Figure 1A).

In the open-field test, *Slc6a20a*^+/–^ and *Slc6a20a^–/–^* mice (3–6 months) showed locomotor activities comparable to those of wild-type (WT) mice (Figure 1B), suggestive of normal locomotor activity in a novel environment. In the Laboras test, where mouse movements were monitored for 4 consecutive days (Quinn et al., 2003, 2006), *Slc6a20a*^+/–^ mice showed normal locomotor activity comparable to that of WT mice (Figure 1C). In contrast, *Slc6a20a^–/–^* mice showed moderately increased locomotor activity during the total period and the light-on period, but not during the light-off period (Figure 1C), suggesting that homozygous but not heterozygous *Slc6a20a* deletion in mice leads to hyperactivity in a familiar environment. The increased activity during the light-on period may also suggest that sleep was disturbed in *Slc6a20a^–/–^* mice, as reported previously in mice lacking the receptor tyrosine phosphatase PTPRS and showing disturbed sleep behaviors and rhythms (Park et al., 2020).

*Slc6a20a*^+/–^ and *Slc6a20a^–/–^* mice spent normal amounts of time in the center region of the open-field arena (Figure 1B), suggesting that anxiety-like behavior was not altered. Similarly, *Slc6a20a*^+/–^ and *Slc6a20a^–/–^* mice showed normal levels of anxiety-like behaviors in the elevated plus-maze and light-dark tests (Figures 1D,E).

These results collectively suggest that homozygous but not heterozygous *Slc6a20a* deletion in mice induces moderate hyperactivity in a familiar environment, without altering anxiety-like behavior.

### *Slc6a20a* Deficiency in Mice Moderately Enhances Repetitive Behavior Without Affecting Social Behavior

**FIGURE 2:**
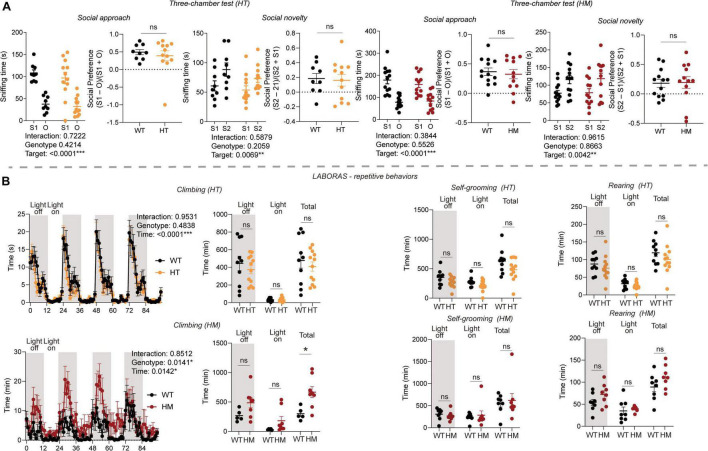
*Slc6a20a* deficiency in mice moderately enhances repetitive behavior without affecting social behavior. **(A)** Normal levels of social approach and social novelty recognition are seen in *Slc6a20a*^+/–^ and *Slc6a20a^–/–^* mice (3–6 months) in the three-chamber test, as shown by the time spent exploring/sniffing a social stranger (S1) vs. an object (O) for social approach or a novel stranger (S2) vs. a familiar stranger (S1) for social novelty recognition, and also by the social preference index derived from sniffing time for S1 – O/sniffing time for S1 + O (or sniffing time for S2 – S1/sniffing time for S2 + S1). (*n* = 9 mice [WT for HT], 12 [HT], 13 [WT for HM], 12 [HM], two-way RM-ANOVA with Bonferroni test [S1 vs. O, or S2 vs. S1], Student’s *t*-test [preference index]). **(B)** Increased repetitive climbing is seen in *Slc6a20a^–/–^* but not *Slc6a20a*^+/–^ mice in the Laboras test, where mouse movements were measured for 4 consecutive days. Note that *Slc6a20a^–/–^* mice show increased repetitive climbing during the total period but not during the light-off/on period, and that *Slc6a20a^–/–^* mice show normal levels of repetitive self-grooming and rearing. (*n* = 10 mice [WT for HT], 12 [HT], 8 [WT for HM], 8 [HM], two-way RM-ANOVA [climbing across 4 days] and Student’s *t*-test [climbing/self-grooming/rearing time]). Statistical significance and *p* values in the graphs; *p < 0.05, **p < 0.01, ***p < 0.001, ns, not significant.

We next subjected *Slc6a20a*-mutant mice to behavioral tests in the social and repetitive behavioral domains. In the three-chamber social-interaction test, *Slc6a20a*^+/–^ and *Slc6a20a^–/–^* mice displayed normal levels of social approach, as shown by time spent exploring social and non-social targets and the social preference index (Figure 2A). In addition, these mice displayed normal levels of social novelty recognition, as shown by time spent exploring novel and familiar social targets (Figure 2A).

In the Laboras test, *Slc6a20a^–/–^* but not *Slc6a20a*^+/–^ mice showed increased repetitive climbing (Figure 2B), a form of repetitive behavior characterized by overhanging from the wire cage lids (Protais et al., 1976; Riffee et al., 1979; Wilcox et al., 1979; Cabib and Puglisi-Allegra, 1985). However, *Slc6a20a*^+/–^ and *Slc6a20a^–/–^* mice did not show any alteration in other repetitive behaviors, such as self-grooming and rearing (Figure 2B). Other behaviors, such as drinking and eating, were also normal in *Slc6a20a^–/–^* and *Slc6a20a*^+/–^ mice (Supplementary Figure 1).

These results suggest that *Slc6a20a* deficiency in mice induces a moderate increase in repetitive behavior without affecting social behaviors, with repetitive climbing but not other repetitive behaviors increased in *Slc6a20a^–/–^* but not *Slc6a20a*^+/–^ mice.

### *Slc6a20a* Deficiency in Mice Moderately Enhances Spatial and Fear Learning and Memory

Because NMDARs critically regulate various forms of learning and memory (Collingridge, 1987; Bliss et al., 2014), we next subjected *Slc6a20a*^+/–^ and *Slc6a20a^–/–^* mice to spatial and fear learning and memory paradigms.

**FIGURE 3:**
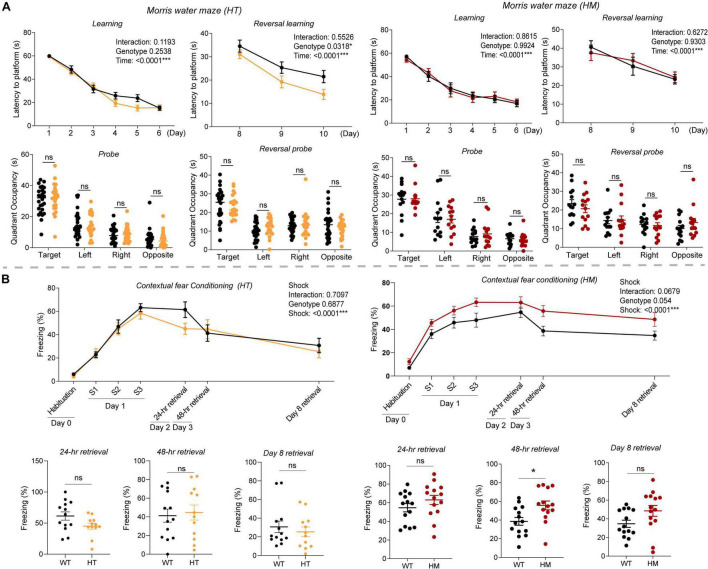
*Slc6a20a* deficiency in mice moderately enhances spatial and fear memory. **(A)** *Slc6a20a*^+/–^ and *Slc6a20a^–/–^* mice (3–6 months) show normal levels of spatial memory acquisition and retrieval (probe test) during the initial phase of the Morris water maze test, whereas *Slc6a20a*^+/–^ but not *Slc6a20a^–/–^* mice show enhanced spatial memory acquisition but not retrieval during the reversal phase of the test. (*n* = 26 mice [WT for HT], 23 [HT], 14 [WT for HM],14 [HM], two-way RM-ANOVA [latency to platform] and Student’s *t*-test [quadrant occupancy]). **(B)** *Slc6a20a*^+/–^ and *Slc6a20a^–/–^* mice (3–6 months) show differential levels of fear memory acquisition (day 1) and retrieval (probe tests; days 2, 3, and 8) in the contextual fear conditioning test. (*n* = 13 mice [WT for HT], 12 [HT], 14 [WT for HM], 14 [HM], two-way RM-ANOVA [acquisition] and Student’s *t*-test [retrievals]). Statistical significance and p values in the graphs; *p < 0.05, ***p < 0.001, ns, not significant.

In the Morris water maze test, *Slc6a20a*^+/–^ and *Slc6a20a^–/–^* mice showed normal levels of spatial memory acquisition and retrieval during the initial learning and probe phases of the test, respectively, compared with WT mice (Figure 3A). In the reversal test, *Slc6a20a*^+/–^ but not *Slc6a20a^–/–^* mice showed enhancements during the acquisition but not probe phase, compared with WT mice (Figure 3A).

In the contextual fear memory conditioning test, *Slc6a20a*^+/–^ mice showed normal levels of fear memory acquisition on day 1 compared with WT mice (Figure 3B). In memory retrieval tests consecutively performed on day 2 (for 24-h retrieval), day 3 (for 48-h retrieval), and day 8 (7-day retrieval), *Slc6a20a*^+/–^ mice showed largely normal fear memory retrieval.

*Slc6a20a^–/–^* mice showed normal levels of fear memory acquisition on day 1 (Figure 3B), although there was an increasing tendency. In the retrieval tests, *Slc6a20a^–/–^* mice showed increased retrieval on day 3 (48-h retrieval); the levels of retrieval on day 2 (24-h retrieval) and day 8 (7-day retrieval) were normal but showed increasing tendencies.

These results collectively suggest that *Slc6a20a* deficiency in mice induces moderate increases in spatial learning and memory in the Morris water maze test and fear learning and memory in contextual fear conditioning test.

### Differentially Expressed Genes (DEGs) in *Slc6a20a*^+/–^ and *Slc6a20a^–/–^* Mice

**FIGURE 4:**
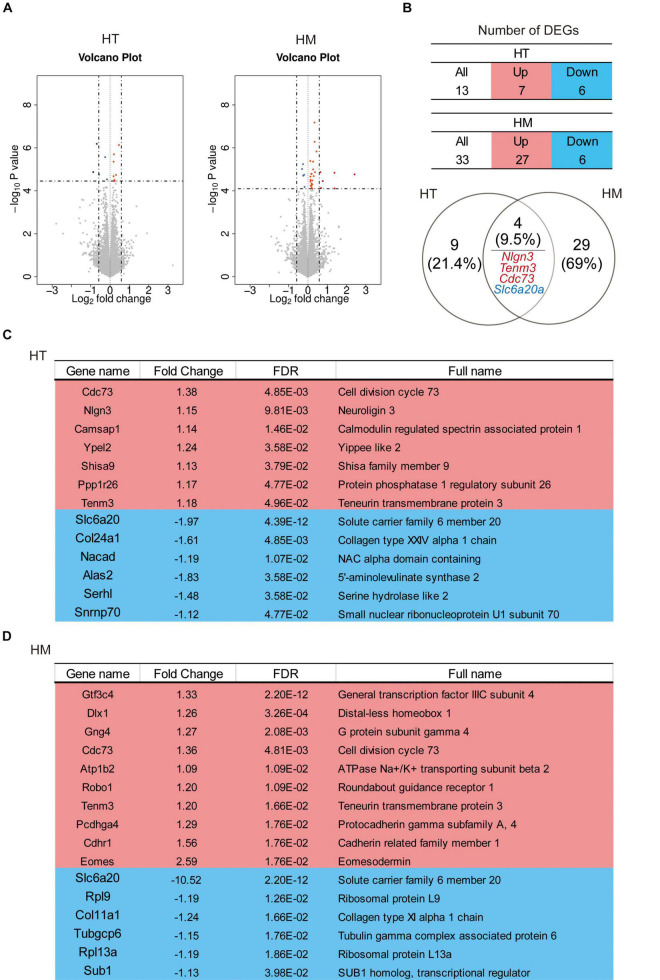
Differentially expressed genes (DEGs) in *Slc6a20a*^+/–^ and *Slc6a20a^–/–^* mice. **(A)** Volcano plot for DEGs from *Slc6a20a*^+/–^ and *Slc6a20a^–/–^* mice (∼P120). (*n* = 4 mice for WT-HT, HT, WT-HM, and HM; dotted lines indicate adjusted *p*-value < 0.05 or | FC| > 1.5; DEGs were defined by adjusted p-values but not fold changes). See Supplementary Table 2 for raw RNA-seq results. **(B)** Summary tables showing the numbers of up- and downregulated DEGs, and Venn diagrams showing the overlap between DEGs from *Slc6a20a*^+/–^ and *Slc6a20a^–/–^* mice. Upregulated, overlapped DEGs (*Nlgn3*/neuroligin-3,*Tenm3*/teneurin-3, and *Cdc73*) and downregulated, overlapped DEGs (*Slc6a20a*) are indicated in red and blue colors, respectively. **(C,D)** Lists of all significantly up- and downregulated DEGs from *Slc6a20a*^+/–^ and *Slc6a20a^–/–^* mice; only the top 10 upregulated DEGs out of 27 are shown for *Slc6a20a^–/–^* mice to save space (see Supplementary Table 3 for further details).

To investigate the molecular phenotypes of *Slc6a20a*^+/–^ and *Slc6a20a^–/–^* mice, we performed RNA-sequencing (RNA-seq) analyses using whole-brain lysates obtained at ∼P120. Our analysis identified 13 DEGs (7 upregulated and 6 downregulated; cutoff, p value < 0.05) in *Slc6a20a*^+/–^ mice and 33 DEGs (27 upregulated and 6 downregulated) in *Slc6a20a^–/–^* mice, with four DEGs overlapping between the genotypes (*Nlgn3*/neuroligin-3, *Tenm3*/teneurin-3, and *Cdc73* upregualted and *Slc6a20a* downregulated) (Figures 4A,B and Supplementary Table 2).

In DEGs from *Slc6a20a*^+/–^ mice, the seven significantly upregulated genes were *Cdc73* (cell division cycle 73), *Nlgn3* (neuroligin 3), *Camsap1* (calmodulin regulated spectrin-associated protein 1), *Ypel2* (Yippee like 2), *Shisa9* (Shisa family member 9), *Ppp1r26* (protein phosphatase 1 regulatory subunit 26), and *Tenm3* (teneurin transmembrane protein 3) (Figure 4C and Supplementary Table 3). The products of some of these genes have been associated with synaptic and neuronal functions. Neuroligin-3 is a synaptic adhesion molecule involved in synapse development and regulation and ASD-related brain functions and behaviors (Etherton et al., 2011; Foldy et al., 2013; Jaramillo et al., 2014; Rothwell et al., 2014; Zhang and Sudhof, 2016; Cao et al., 2018; Sudhof, 2018). CAMSAP1 binds to the minus end of microtubules and regulates neuronal polarity/migration and cortical lamination (Meng et al., 2008; Akhmanova and Steinmetz, 2015; Zhou et al., 2020). Shisa9 (also known as CKAMP44) is an AMPA receptor auxiliary protein that regulates the trafficking, subcellular localization, and function of AMPA receptors (von Engelhardt et al., 2010; Jacobi and von Engelhardt, 2018; von Engelhardt, 2019). Teneurin-3 is a transmembrane protein involved in homophilic adhesion as well as heterophilic adhesion with latrophilins that regulates synapse specificity and neural circuit assembly (Mosca et al., 2012; Li et al., 2018; Sando et al., 2019; Pederick and Luo, 2021).

The six significantly downregulated genes in *Slc6a20a*^+/–^ mice were *Slc6a20a* (solute carrier family 6 member 20), as expected, followed by *Col24a1* (collagen type XXIV alpha 1 chain), *Nacad* (NAC alpha domain-containing), *Alas2* (5′-aminolevulinate synthase 2), and *Serhl2* (serine hydrolase-like 2), and *Snrnp70* (small nuclear ribonucleoprotein U1 subunit 70) (Figure 4C). Interestingly, Alas2 is an enzyme localized in the mitochondria of erythrocytes; it regulates the heme biosynthetic pathway and is implicated in X-linked sideroblastic anemia (XLSA) (Nzelu et al., 2021).

In *Slc6a20a^–/–^* mice, the top ten upregulated genes were *Gtf3c4* (general transcription factor IIIC subunit 4), *Dlx1* (distal-less homeobox 1), *Gng4* (G protein subunit gamma 4), *Cdc73* (cell division cycle 73; also upregulated in *Slc6a20a*^+/–^ mice), *Atp1b2* (ATPase Na + /K + transporting subunit beta 2), *Robo1* (roundabout guidance receptor 1), *Tenm3* (teneurin transmembrane protein 3), *Pcdhga4* (protocadherin gamma subfamily A, 4), *Cdhr1* (cadherin related family member 1), and *Eomes* (eomesodermin) (Figure 4D). Dlx1 is a homeobox transcription factor that regulates neuronal differentiation (Petryniak et al., 2007; Lee B. et al., 2018; Pla et al., 2018; Lindtner et al., 2019). Atp1b2 is a sodium-potassium ATPase that regulates neuronal excitability (Larsen et al., 2016). Robo1 is a transmembrane protein that regulates axon guidance and neuronal migration (Seeger et al., 1993; Kidd et al., 1998).

Many DEGs from *Slc6a20a^–/–^* mice not mentioned above have also been associated with synaptic and neuronal functions; examples include *Mdga1* (MAM domain containing glycosylphosphatidylinositol anchor 1) (Lee et al., 2013; Pettem et al., 2013; Kim et al., 2017; Um and Ko, 2017), *Cacng8* (calcium voltage-gated channel auxiliy subunit gamma 8) (Nicoll et al., 2006; Diaz-Alonso and Nicoll, 2021), *Sema5a* (semaphorin 5A) (Duan et al., 2014), and *Nlgn3* (neuroligin 3) (Sudhof, 2018). It is also notable that neuroligin-3 and teneurin-3 are similarly upregulated in *Slc6a20a*^+/–^ and *Slc6a20a^–/–^* mice, suggesting that their upregulations may represent a shared mechanism responding to *Slc6a20a* deletion. Lastly, the six significantly downregulated genes in *Slc6a20a^–/–^* mice were *Slc6a20a, Rpl9* (ribosomal protein L9), *Col11a1* (collagen type XI alpha 1 chain), *Tubgcp6* (tubulin gamma complex associated protein 6), *Rpl13a* (ribosomal protein L13A), and *Sub1* (SUB1 homolog, transcriptional regulator) (Figure 4D).

These results collectively suggest that both *Slc6a20a*^+/–^ and *Slc6a20a^–/–^* mice show upregulation of genes that are associated with synaptic and neuronal functions and downregulation of genes associated with ribosomal and mitochondrial functions. Similar results were obtained from our gene set enrichment analysis (GSEA; see below).

### Biological Functions Altered in the Transcriptomes of *Slc6a20a*^+/–^ and *Slc6a20a^–/–^* Mice Revealed by GSEA

Since our DEG analysis yielded small numbers of DEGs, we performed gene set enrichment analysis (GSEA), which can identify altered biological functions using a large number of small but coordinated transcriptomic changes in a less biased manner than analysis of a small number of large transcriptomic changes (Subramanian et al., 2005, 2007).

**FIGURE 5:**
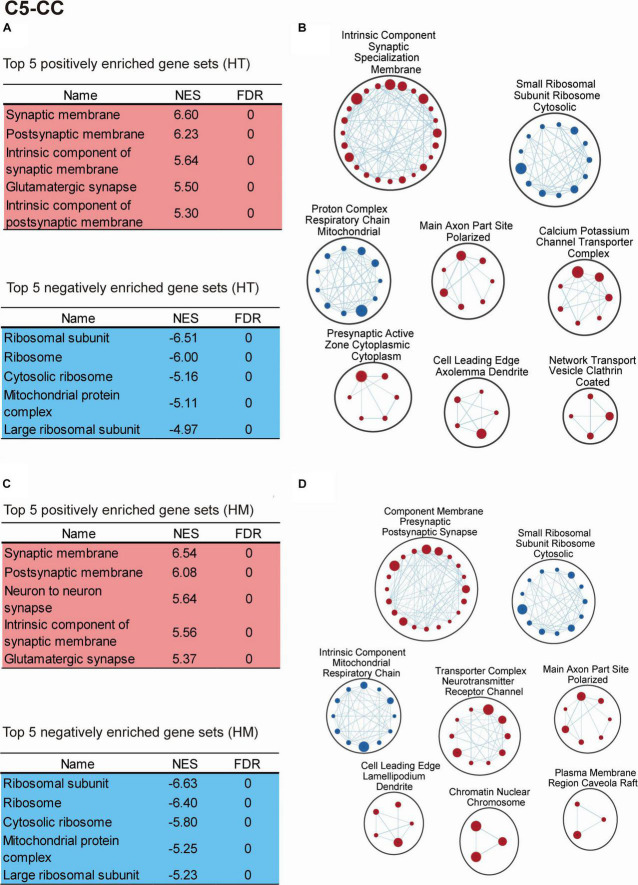
Biological functions altered in the transcriptomes of *Slc6a20a*^+/–^ and *Slc6a20a^–/–^* mice, as revealed by GSEA. **(A–D)** GSEA results for transcriptomes from WT and *Slc6a20a*^+/–^ mice (HT/WT transcripts) and WT and *Slc6a20a^–/–^* mice (HM/WT transcripts), as shown by the top five most strongly enriched gene sets **(A,C)** and clustering of the enriched gene sets using CytoScape EnrichmentApp **(B,D)**. See Supplementary Table 4 for enriched gene sets beyond the top five genes shown in the table. Gene set clusters composed of > 3 gene sets are shown. These GSEA results were derived using the gene sets of the cellular component (CC) domain in the C5 database; see Supplementary Figures 2,3 for the GSEA results from the biological process (BP) and molecular function (MF) domains in the C5 database. NES, normalized enrichment score; FDR, false detection rate. [*n* = 4 mice for WT-HT, HT, WT-HM, and HM; FDR < 0.05 **(B,D)**].

The transcripts derived from WT and *Slc6a20a*^+/–^ mice (HT/WT transcripts; ∼P120 whole brain) were positively enriched for gene sets associated with neuronal synapses, as shown by the top five enriched gene sets (Figure 5A and Supplementary Table 4). Clustering of the positively enriched gene sets using CytoScape Enrichment App (Merico et al., 2010; Isserlin et al., 2014) further confirmed that there were positive enrichments for functions associated with neuronal synapses, such as synaptic specialization, presynaptic active zone, ion channels, dendrites, and axons (Figure 5B).

These results were obtained using the gene sets of the cellular component domain in the C5 database; we also obtained similar results using the gene sets of the biological process and molecular function domains of the C5 database (Supplementary Figures 2A,B, 3A,B and Supplementary Table 4). In addition, the use of gene sets in the KEGG domain indicated positive enrichments for synapse-related gene sets including long-term potentiation and gap junction, known to cooperate with excitatory synapses (Lee et al., 2021), as well as negative enrichments for ribosome/mitochondria-related gene sets (Supplementary Figures 4A,B and Supplementary Table 4).

The HT/WT transcripts were negatively enriched for gene sets associated with ribosomes and mitochondria (Figure 5A and Supplementary Table 4). In addition, CytoScape Enrichment App analysis revealed similar negative enrichments for ribosome/mitochondria-related functions, such as ribosomal subunits and respiratory chain complex (Figure 5B).

The transcripts derived from WT and *Slc6a20a^–/–^* mice (HM/WT transcripts) were positively enriched for gene sets associated with neuronal synapses, as supported by the top five gene sets and CytoScape Enrichment App clustering (Figures 5C,D and Supplementary Table 4). In addition, the HM/WT transcripts were negatively enriched for gene sets associated with ribosomes and mitochondria, as supported by the top five gene sets and CytoScape Enrichment App clustering (Figures 5C,D and Supplementary Table 4). Similar results were obtained using the gene sets of the biological process and molecular function domains (Supplementary Figures 2C,D, 3C,D and Supplementary Table 4).

These results collectively suggest that heterozygous and homozygous deletion of *Slc6a20a* in mice leads to similar increases in synapse-associated genes and similar decreases in ribosome- and mitochondria-related genes.

### ASD-Related Transcriptomic Changes in *Slc6a20a*^+/–^ and *Slc6a20a^–/–^* Mice Revealed by GSEA

Previous studies investigated transcriptomic changes associated with ASD (Garbett et al., 2008; Voineagu et al., 2011; Gupta et al., 2014; Parikshak et al., 2016; Velmeshev et al., 2019) and reported gene sets that are up- or downregulated in ASD (termed ASD-related gene sets hereafter), including DEG_Up_Voineagu, Co-Exp_Up_M16_Voineagu, DEG_Down_Voineagu, and Co-Exp_Down_M12_Voineagu (Voineagu et al., 2011; Werling et al., 2016) (details on these gene sets are summarized in Supplementary Table 5).

In addition, a large number of previous studies on ASD led to the compilation of ASD-risk gene sets, including SFARI genes (all genes and high-confidence category 1 genes) (Abrahams et al., 2013)^1^, FMRP targets (Darnell et al., 2011; Werling et al., 2016), De Novo Missense (protein-disrupting or missense rare *de novo* variants) (Iossifov et al., 2014; Werling et al., 2016), De Novo Variants (protein-disrupting rare *de novo* variants) (Iossifov et al., 2014; Werling et al., 2016), and AutismKB (Autism KnowledgeBase) (Xu et al., 2012; Yang et al., 2018; Supplementary Table 5). The genes in these ASD-risk gene sets are thought be generally downregulated in ASD because many of the mutations are missense, nonsense, splice-site, frame-shift, and deletion mutations.

**FIGURE 6:**
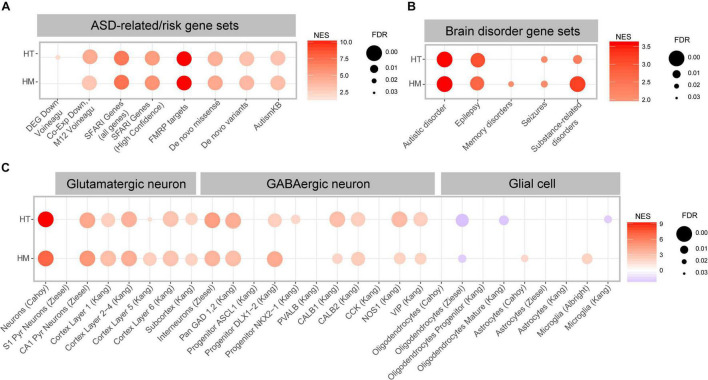
ASD-related transcriptomic changes in *Slc6a20a*^+/–^ and *Slc6a20a^–/–^* mice are revealed by GSEA. **(A)** GSEA results for transcriptomes from WT and *Slc6a20a*^+/–^ mice (HT/WT transcripts) and WT and *Slc6a20a^–/–^* mice (HM/WT transcripts) for ASD-related gene sets (DEG_Down_Voineagu and Co-Exp_Down_M12_Voineagu) and ASD-risk gene sets (SFARI genes (all genes and high-confidence category 1 genes), FMRP targets, De Novo Missense, De Novo Variants, and AutismKB). See Supplementary Table 4 for further details on the GSEA results. NES, normalized enrichment score; FDR, false detection rate. (n = 4 mice for WT-HT, HT, WT-HM, and HM). **(B)** GSEA results for transcriptomes from WT and *Slc6a20a*^+/–^ mice (HT/WT transcripts) and WT and *Slc6a20a^–/–^* mice (HM/WT transcripts) for gene sets in the DisGeNet (https://www.disgenet.org/). See Supplementary Table 4 for further details of the GSEA results. NES, normalized enrichment score; FDR, false detection rate. (n = 4 mice for WT-HT, HT, WT-HM, and HM). **(C)** GSEA results for transcriptomes from WT and *Slc6a20a*^+/–^ mice (HT/WT transcripts) and WT and *Slc6a20a^–/–^* mice (HM/WT transcripts) for cell-type-specific gene sets. See Supplementary Table 4 for further details of the GSEA results. NES, normalized enrichment score; FDR, false detection rate. (*n* = 4 mice for WT-HT, HT, WT-HM, and HM).

Using these gene sets, we performed GSEA using the HT/WT and HM/WT transcripts from *Slc6a20a*-mutant mice. The HT/WT and HM/WT transcripts were positively and similarly enriched for ASD-related gene sets that are downregulated in ASD, such as DEG_Down_Voineagu and Co-Exp_Down_M12_Voineagu (Figure 6A and Supplementary Table 4), suggesting that both HT/WT and HM/WT transcripts exhibit patterns opposite those observed in ASD (termed anti-ASD hereafter).

The HT/WT and HM/WT transcripts were also positively and similarly enriched for all of the tested ASD-risk gene sets, including SFARI genes, FMRP targets (most strongly enriched), De Novo Missense, De Novo Variants, and AutismKB (Figure 6A and Supplementary Table 4), and thus again exhibited an anti-ASD pattern.

When tested against gene sets associated with various brain disorders (DisGeNet^2^ (Pinero et al., 2017, 2020), the HT/WT and HM/WT transcripts were positively and similarly enriched for ASD and epilepsy-related gene sets, and the HM/WT transcripts were positively and strongly enriched for substance use-related gene sets relative to the HT/WT transcripts (Figure 6B and Supplementary Table 4).

Distinct cell-type-specific transcriptomic changes have also been reported in ASD, including downregulation of neuron- and oligodendrocyte-related genes and upregulation of astrocyte- and microglia-related genes (Voineagu et al., 2011; Werling et al., 2016). This led us to test if the HT/WT and HM/WT transcripts are enriched in these previously reported cell type-specific gene sets (Albright and Gonzalez-Scarano, 2004; Cahoy et al., 2008; Kang et al., 2011; Zeisel et al., 2015; Werling et al., 2016; Velmeshev et al., 2019, 2020; Supplementary Table 5).

The HT/WT and HM/WT transcripts were positively enriched for gene sets associated with glutamate and GABA neurons (Figure 6C and Supplementary Table 4), suggestive of anti-ASD transcriptomic patterns. However, the HT/WT and HM/WT transcripts were negatively and moderately enriched for gene sets associated with oligodendrocytes (Figure 6C), suggestive of “ASD-like” or “pro-ASD” transcriptomic patterns. Intriguingly, the HT/WT and HM/WT transcripts displayed weak enrichments for astrocyte and microglia-related gene sets relative to those for glutamate/GABA neurons; thus we observed both positive and negative enrichments for microglia (Figure 6C).

These results collectively suggest that heterozygous and homozygous *Slc6a20a* deletions in mice lead to largely anti-ASD transcriptomic enrichment patterns, as supported by the enrichment patterns for ASD-related/risk and cell-type-specific gene sets. In addition, the cell-type-specific transcriptomic changes induced by *Slc6a20a* deletion are stronger in neurons and oligodendrocytes relative to glial cells.

## Discussion

In the present study, we analyzed the behavioral and transcriptomic phenotypes of *Slc6a20a*^+/–^ and *Slc6a20a^–/–^* mice. Our results revealed that these mice display differential hyperactivity, repetitive climbing, and moderately enhanced spatial and fear memory, as well as upregulation of synapse-related genes and downregulation of ribosome- and mitochondria-related genes. ASD-related transcriptomic changes are also observed, such as upregulation of ASD-related genes, including FMRP targets, and stronger upregulation of neuron-related genes compared to glia-related genes.

The behavioral phenotypes seem to be stronger in *Slc6a20a^–/–^* mice than in *Slc6a20a*^+/–^ mice in some assays. For instance, *Slc6a20a^–/–^* mice are hyperactive in a familiar environment (Laboras results) and show increased self-grooming in Laboras cages and abnormally increased 48-hr fear memory, whereas *Slc6a20a*^+/–^ mice do not exhibit such changes. However, spatial learning and memory is increased in *Slc6a20a*^+/–^ but not in *Slc6a20a^–/–^* mice during the reversal but not the initial phase of the Morris water maze test. These results collectively suggest that heterozygous and homozygous *Slc6a20a* deletions in mice lead to differential behavioral phenotypes.

The mechanisms underlying these behavioral deficits are unclear. However, the RNA-seq results obtained from *Slc6a20a*^+/–^ and *Slc6a20a^–/–^* mice indicate that synapse-related genes are upregulated. We note that *Slc6a20a*^+/–^ mice have been shown to display increased NMDAR function at juvenile stages (Bae et al., 2021). In addition, the increased *Slc6a20a* expression in *Pten*-mutant mice has been causally associated with decreased ambient glycine levels and NMDAR function, and increased repetitive self-grooming (Bae et al., 2021). Moreover, neuroligin-3, which is upregulated in both *Slc6a20a*^+/–^ and *Slc6a20a^–/–^* mice, has been associated with the regulation of NMDAR functions (Etherton et al., 2011; Polepalli et al., 2017; Zhang et al., 2017). It is thus tempting to speculate that altered NMDAR function in *Slc6a20a*^+/–^ and *Slc6a20a^–/–^* mice might be associated with the behavioral deficits observed in these animals.

It should be noted that Slc6a20a expression at the protein level is much higher at late embryonic and early postnatal stages than at adult stages, while the behavioral experiments were mainly performed using mutant mice at ages around 3-6 months. Therefore, it is less likely that adult-stage deficiency of Slc6a20a directly causes the behavioral deficits, and the mechanistic deviations occurred at early developmental stages may have long-lasting impacts. Details on such mechanisms remain to be determined. However, given that Slc6a20a deficiency leads to NMDAR hyperfunction at juvenile stages (Bae et al., 2021), as mentioned above, and NMDAR function has profound impacts on the brain development and function across all developmental stages (Paoletti et al., 2013; Zhu and Paoletti, 2015; Hansen et al., 2018), it is probable that the NMDAR function impaired during early development have long-lasting effects on neuronal and synapse/circuit mechanisms that are associated with the observed behavioral deficits.

A notable transcriptomic change associated with the increased synaptic gene expression seen in *Slc6a20a*^+/–^ and *Slc6a20a^–/–^* mice is the decreased expression levels of ribosomal and mitochondrial genes. This leads us to question whether ribosomal and mitochondrial gene downregulation are causally associated with the synaptic gene upregulation and behavioral deficits in the mutant mice. It has been shown that synaptic proteins are reciprocally related to protein synthesis in ASD (Santini and Klann, 2014). In addition, mitochondrial dysfunction has been linked to synaptic deficits (Li et al., 2004; Vos et al., 2010; Sheng and Cai, 2012; Lee A. et al., 2018) and ASD (Hollis et al., 2017; Frye, 2020; Rojas-Charry et al., 2021), and ribosomal dysfunction has been associated with ASD and epilepsy (Lombardo, 2021). These results suggest that ribosomal and mitochondrial gene downregulation observed in our mutant mice may contribute to their synaptic and behavioral deficits.

GSEA using brain disorder-related gene sets shows that the *Slc6a20a*^+/–^ and *Slc6a20a^–/–^* transcriptomes are associated with ASD and epilepsy, and that the *Slc6a20a^–/–^* transcriptome is more strongly associated with substance use-related disorders than the *Slc6a20a*^+/–^ transcriptome. Among the ASD-related gene sets, the *Slc6a20a*^+/–^ and *Slc6a20a^–/–^* transcriptomes are more strongly enriched for FMRP targets associated with the fragile X syndrome. These results suggest that *Slc6a20a* deletion in mice leads to transcriptomic changes associated with multiple brain disorders. In addition, given that many FMRP targets have been related to synaptic functions (Bagni and Zukin, 2019), these results further support the hypothesis that synaptic deficits may underlie the behavioral deficits in these mutant mice.

GSEA using cell-type-specific gene sets indicates that the *Slc6a20a*^+/–^ and *Slc6a20a^–/–^* transcriptomes are more strongly enriched for neuron (glutamate and GABA)-related gene sets relative to glia (astrocyte/microglia)-related gene sets. *Slc6a20a* has been shown to be expressed more strongly in glial cells (astrocytes and microglia) than in neurons (glutamate and GABA) (Bae et al., 2021). Our results suggest that the transcriptomic changes observed in synapse/ribosome/mitochondria-related genes in the mutant brain likely represent changes occurring in neurons rather than cell-autonomous changes occurring in glial cells (astrocytes and microglia).

Lastly, it should be pointed out that the differences between *Slc6a20a*^+/–^ and *Slc6a20a^–/–^* mice were greater in behavioral phenotypes than in transcriptomic phenotypes. It is possible that the subtle differences between *Slc6a20a*^+/–^ and *Slc6a20a^–/–^* mice in the expression levels of neuron/glia-related genes (i.e., different cortical layers, GABA subtypes, and glial cell types) may still contribute to the differential behavioral changes. Alternatively, the transcriptomic changes may reflect compensatory changes initiated in an effort to rescue the gene deletion effects, rather than those mediating the pathophysiological changes. Further validation of the mechanistic/functional changes suggested by the transcriptomic changes should be tested at the protein, synaptic, and functional levels.

In summary, we herein show that heterozygous and homozygous *Slc6a20a* deletions in mice lead to differential behavioral deficits in locomotor, repetitive behavioral, and spatial and fear memory domains and transcriptomic changes in genes associated with synapses, ribosomes, mitochondria, ASD, epilepsy, and neurons.

## Data Availability Statement

The RNA-Seq data presented in the study are deposited in the Gene Expression Ombinus (GEO) repository at the National Center for Biotechnology Information (NCBI), accession number GSE193387.

## Ethics Statement

The animal study was reviewed and approved by Committee of Animal Research at KAIST (KA2020-89).

## Author Contributions

JK, JR, and MB performed behavioral experiments. SK performed immunoblot analysis. HK performed RNA-Seq analyses. MB, HK, and EK wrote the manuscript. All authors contributed to the article and approved the submitted version.

## Conflict of Interest

The authors declare that the research was conducted in the absence of any commercial or financial relationships that could be construed as a potential conflict of interest.

## Publisher’s Note

All claims expressed in this article are solely those of the authors and do not necessarily represent those of their affiliated organizations, or those of the publisher, the editors and the reviewers. Any product that may be evaluated in this article, or claim that may be made by its manufacturer, is not guaranteed or endorsed by the publisher.

## Footnotes

1
https://gene.sfari.org/

2
https://www.disgenet.org/

## Supplementary Material

Supplementary Figure 1 Normal levels of repetitive drinking and eating and immobility and other parameters in *Slc6a20a^–/–^* and *Slc6a20a*^+/–^ mice in the Laboras test. **(A,B)** Normal levels of repetitive drinking and eating are seen for *Slc6a20a^–/–^* and *Slc6a20a*^+/–^ mice in the Laboras test, where mouse movements were measured for 4 consecutive days, as shown by total time spent drinking and eating. (*n* = 10 mice [WT for HT], 12 [HT], 8 [WT for HM], 8 [HM], Student’s *t*-test). Statistical significance and *p* values in the graphs; ns, not significant.

Supplementary Figure 2 Biological functions altered in the transcriptomes of *Slc6a20a*^+/–^ and *Slc6a20a^–/–^* mice, as revealed by GSEA with gene sets in the biological process domain. **(A,B)** GSEA results for transcriptomes from WT and *Slc6a20a*^+/–^ mice (HT/WT transcripts) and WT and *Slc6a20a^–/–^* mice (HM/WT transcripts), using the gene sets in the biological process domain in the C5 database, as shown by top five most strongly enriched gene sets **(A,C)** and clustering of the enriched gene sets using CytoScape EnrichmentApp **(B,D)**. See Supplementary Table 4 for enriched gene sets additional to the top five gene sets shown in the table. Gene set clusters composed of > 3 gene sets are shown. NES, normalized enrichment score; FDR, false detection rate. [*n* = 4 mice for WT-HT, HT, WT-HM, and HM; FDR < 0.05 **(B,D)**].

Supplementary Figure 3 Biological functions altered in the transcriptomes of *Slc6a20a*^+/–^ and *Slc6a20a^–/–^* mice, as revealed by GSEA with gene sets in the molecular function domain. **(A,B)** GSEA results for transcriptomes from WT and *Slc6a20a*^+/–^ mice (HT/WT transcripts) and WT and *Slc6a20a^–/–^* mice (HM/WT transcripts) using the gene sets in the molecular function domain in the C5 database, as shown by top five most strongly enriched gene sets **(A,C)** and clustering of the enriched gene sets using CytoScape EnrichmentApp **(B,D)**. See Supplementary Table 4 for enriched gene sets additional to the top five gene sets shown in the table. Gene set clusters composed of > 3 gene sets are shown. NES, normalized enrichment score; FDR, false detection rate. [*n* = 4 mice for WT-HT, HT, WT-HM, and HM; FDR < 0.05 **(B,D)**].

Supplementary Figure 4 Biological functions altered in the transcriptomes of *Slc6a20a*^+/–^ and *Slc6a20a^–/–^* mice, as revealed by GSEA with gene sets in the KEGG database. **(A,B)** GSEA results for transcriptomes from WT and *Slc6a20a*^+/–^ mice (HT/WT transcripts) and WT and *Slc6a20a^–/–^* mice (HM/WT transcripts) using the gene sets in the KEGG database, as shown by top five most strongly enriched gene sets **(A,C)** and clustering of the enriched gene sets using CytoScape EnrichmentApp **(B,D)**. See Supplementary Table 4 for enriched gene sets additional to the top five gene sets shown in the table. Gene set clusters composed of >3 gene sets are shown. NES, normalized enrichment score; FDR, false detection rate. [*n* = 4 mice for WT-HT, HT, WT-HM, and HM; FDR < 0.05 **(B,D)**].

Supplementary Table 1 Statistical results.

Supplementary Table 2 Raw RNA-Seq results.

Supplementary Table 3 DEGs derived from RNA-Seq results.

Supplementary Table 4 GSEA results.

Supplementary Table 5 Gene sets used in the GSEA analysis.
