# Supplementary figures and images for: Slc6a20a Heterozygous and Homozygous Mutant Mice Display Differential Behavioral and Transcriptomic Changes
Source: Front Mol Neurosci. 2022 Mar 7;15:857820. doi: 10.3389/fnmol.2022.857820 (PMC8936588; doi:10.3389/fnmol.2022.857820)

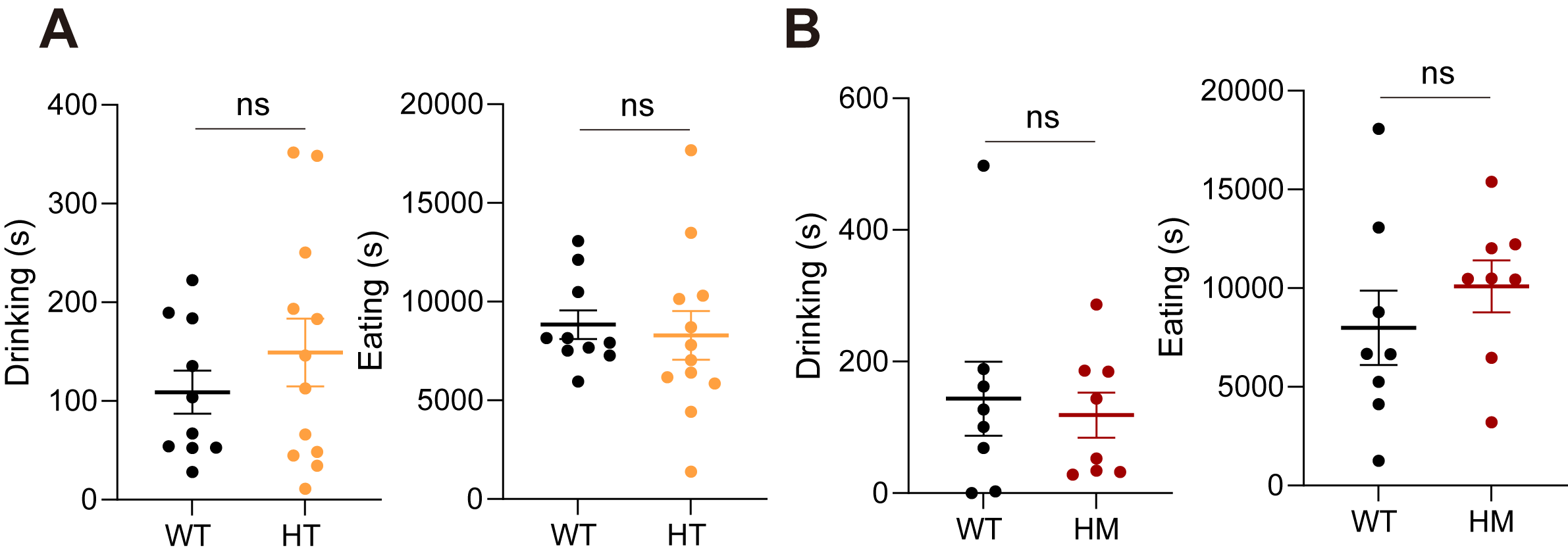

Supplement: Supplementary Figure 1 — Normal levels of repetitive drinking and eating and immobility and other parameters in Slc6a20a–/– and Slc6a20a+/– mice in the Laboras test. (A,B) Normal levels of repetitive drinking and eating are seen for Slc6a20a–/– and Slc6a20a+/– mice in the Laboras test, where mouse movements were measured for 4 consecutive days, as shown by total time spent drinking and eating. (n = 10 mice [WT for HT], 12 [HT], 8 [WT for HM], 8 [HM], Student’s t-test). Statistical significance and p values in the graphs; ns, not significant. [file Image_1.TIF]

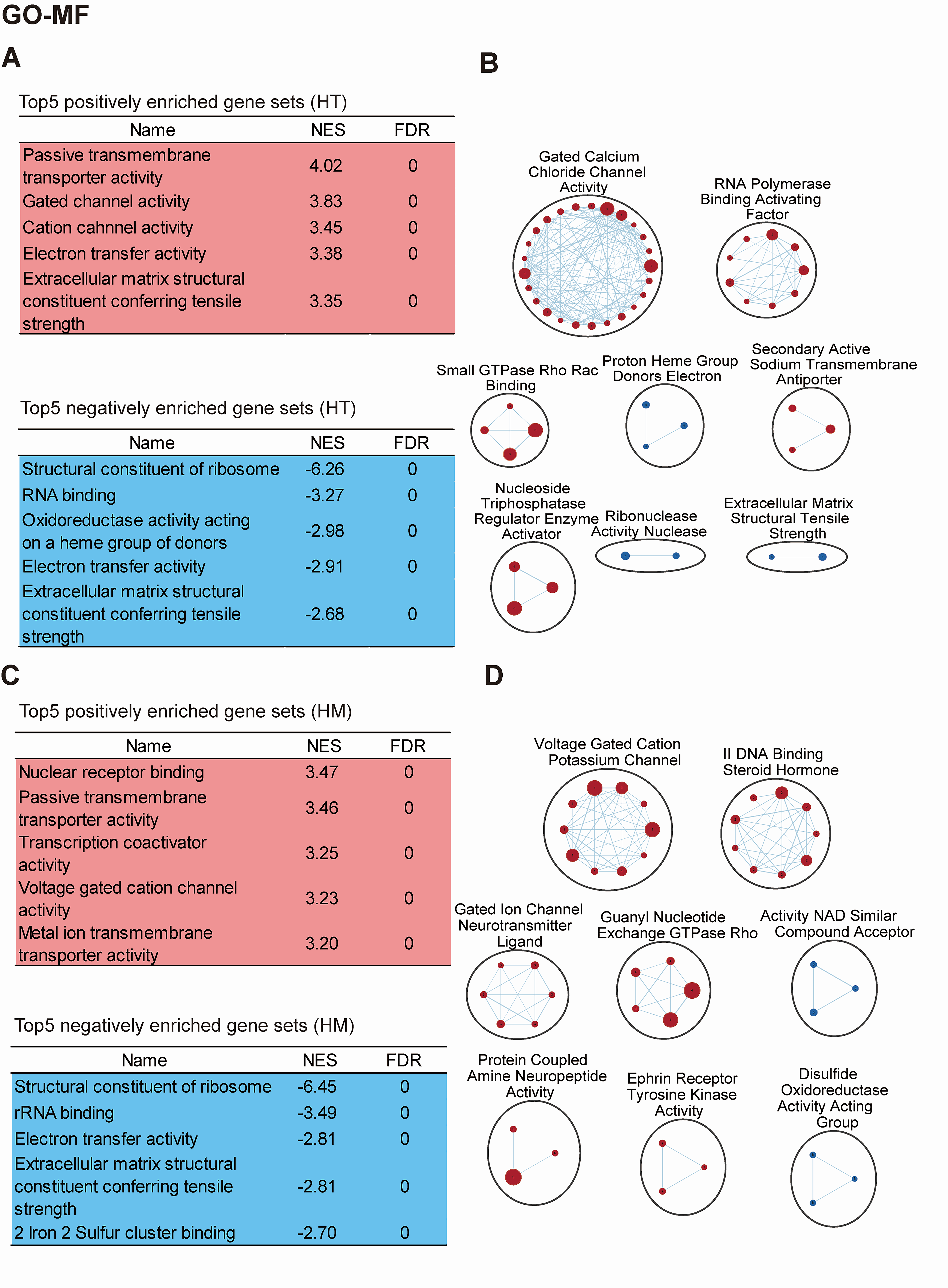

Supplement: Supplementary Figure 2 — Biological functions altered in the transcriptomes of Slc6a20a+/– and Slc6a20a–/– mice, as revealed by GSEA with gene sets in the biological process domain. (A,B) GSEA results for transcriptomes from WT and Slc6a20a+/– mice (HT/WT transcripts) and WT and Slc6a20a–/– mice (HM/WT transcripts), using the gene sets in the biological process domain in the C5 database, as shown by top five most strongly enriched gene sets (A,C) and clustering of the enriched gene sets using CytoScape EnrichmentApp (B,D). See Supplementary Table 4 for enriched gene sets additional to the top five gene sets shown in the table. Gene set clusters composed of > 3 gene sets are shown. NES, normalized enrichment score; FDR, false detection rate. [n = 4 mice for WT-HT, HT, WT-HM, and HM; FDR < 0.05 (B,D)]. [file Image_2.TIF]

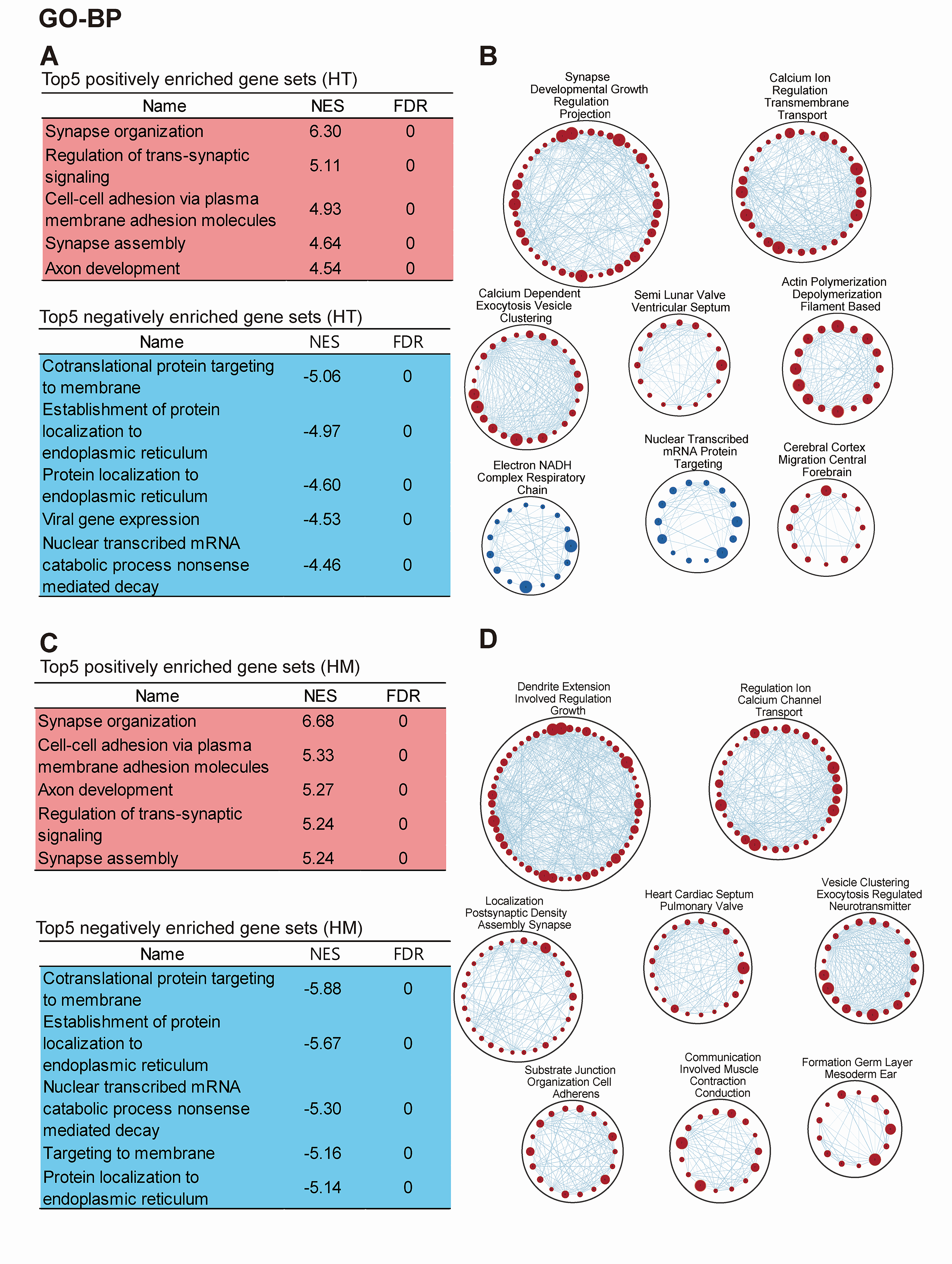

Supplement: Supplementary Figure 3 — Biological functions altered in the transcriptomes of Slc6a20a+/– and Slc6a20a–/– mice, as revealed by GSEA with gene sets in the molecular function domain. (A,B) GSEA results for transcriptomes from WT and Slc6a20a+/– mice (HT/WT transcripts) and WT and Slc6a20a–/– mice (HM/WT transcripts) using the gene sets in the molecular function domain in the C5 database, as shown by top five most strongly enriched gene sets (A,C) and clustering of the enriched gene sets using CytoScape EnrichmentApp (B,D). See Supplementary Table 4 for enriched gene sets additional to the top five gene sets shown in the table. Gene set clusters composed of > 3 gene sets are shown. NES, normalized enrichment score; FDR, false detection rate. [n = 4 mice for WT-HT, HT, WT-HM, and HM; FDR < 0.05 (B,D)]. [file Image_3.TIF]

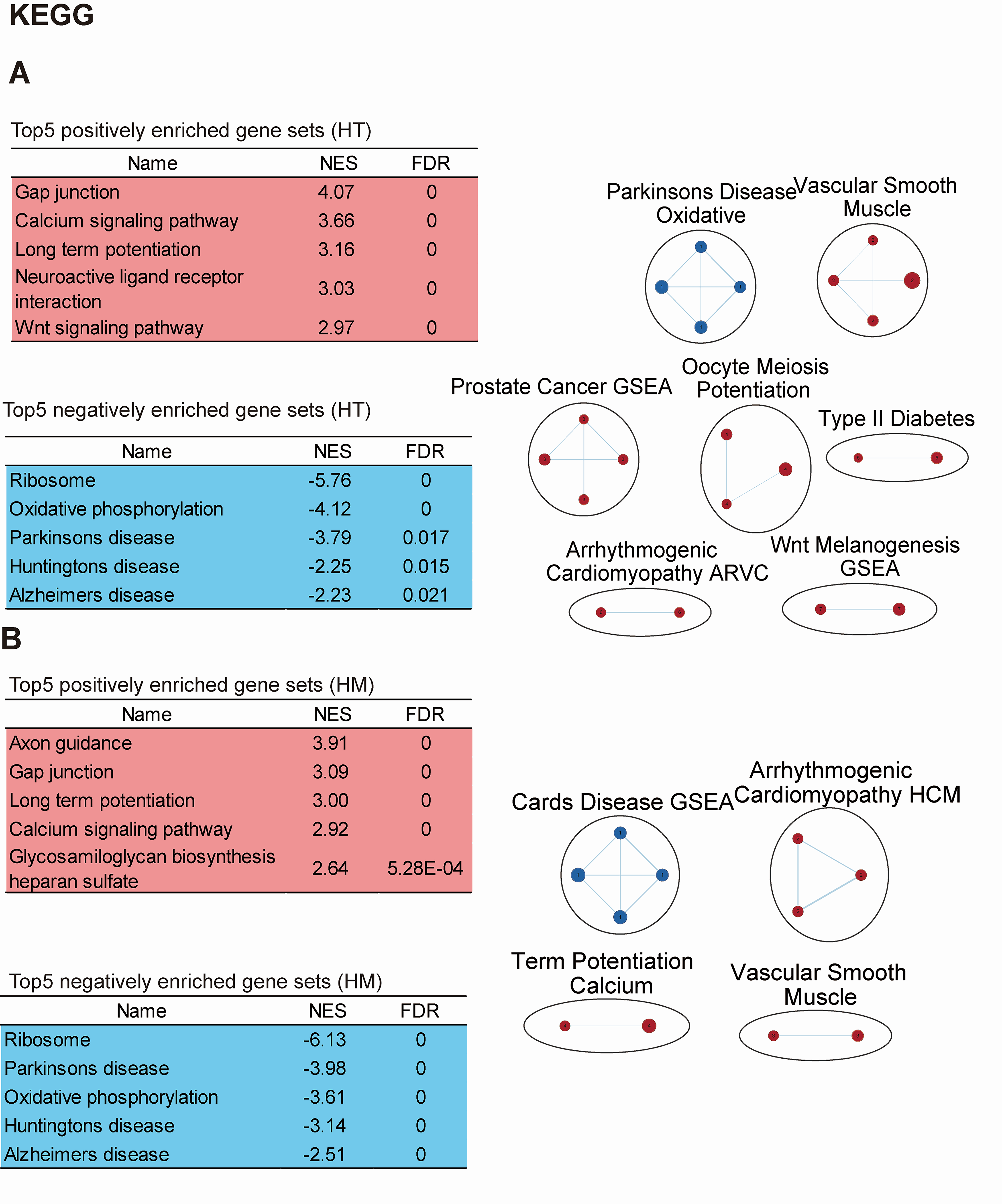

Supplement: Supplementary Figure 4 — Biological functions altered in the transcriptomes of Slc6a20a+/– and Slc6a20a–/– mice, as revealed by GSEA with gene sets in the KEGG database. (A,B) GSEA results for transcriptomes from WT and Slc6a20a+/– mice (HT/WT transcripts) and WT and Slc6a20a–/– mice (HM/WT transcripts) using the gene sets in the KEGG database, as shown by top five most strongly enriched gene sets (A,C) and clustering of the enriched gene sets using CytoScape EnrichmentApp (B,D). See Supplementary Table 4 for enriched gene sets additional to the top five gene sets shown in the table. Gene set clusters composed of >3 gene sets are shown. NES, normalized enrichment score; FDR, false detection rate. [n = 4 mice for WT-HT, HT, WT-HM, and HM; FDR < 0.05 (B,D)]. [file Image_4.TIF]
